# Regeneration of dermal patterns from the remaining pigments after surgery in *Eublepharis macularius* (a case report)

**DOI:** 10.1186/s12917-016-0765-x

**Published:** 2016-07-12

**Authors:** Noriyuki Nakashima

**Affiliations:** Department of Physiology, Graduate School of Medicine, Kyoto University, Yoshida-Konoe, Sakyo-ku, Kyoto, 606-8501 Japan; Department of Physiology, School of Medicine, Kurume University, 67, Asahi-machi, Kurume-shi, Fukuoka 830-0011 Japan

**Keywords:** *Eublepharis macularius*, Dermal scar, Pigment regeneration, Pigment preservation

## Abstract

**Background:**

Dermal injury of the *Eublepharis macularius* (leopard gecko) often results in a loss of the spotted patterns. The scar is usually well recovered, but the spots and the tubercles may be lost depending on the size and part of the lesion. This report presents a surgical attempting, in which the pigments in the edge of the remaining skin flap are partially preserved to maximally restore the natural pigmentation patterns during the course of dermal regeneration.

**Case presentation:**

A four-year-old female lizard *E. macularius* was evaluated due to a subcutaneous tumor in the occipito-pterional portion behind its right eye. A solid tumor beneath the skin was surgically enucleated under general anesthesia. Then, the ulcerated skin was dissected away together with the tumor. The necrotic edge of the remaining skin flap was carefully trimmed to leave as much of the pigmented portions as possible on the outskirt of the skin flap. The scar was covered with the remaining skin flap, and the uncovered lesion was protected with Vaseline containing gentamicin. The lesion was rapidly covered with regenerated dermis within a week, and the epidermis with round and well-oriented pigmented spots were almost completely restored in four months.

**Conclusion:**

The surgical suture of the skin flap after removal of the ulcerated margins resulted in the scar-free regeneration of the scales and the pigmented spots. And the pigmented spots of the remaining skin close to the lesion site might be a source of the regenerated spots.

## Background

*Eublepharis macularius* (*E. macularius*, also known as a leopard gecko) becomes popular since this exotic reptile is docile in nature and is easy to handle; moreover, it is very unique and diverse in its coloration and dermal patterns [1]. Scientifically, *E. macularius* is considered a good model for the study of regeneration, because of its tremendous potential for epimorphic scar-free regeneration at the wound site in the body skin and the tail [2–5]. And pigment pattern regeneration is of interest to both practitioners and researchers [6].

When the loss of the skin tissue of *E. macularius* was large, the lost scales were usually replaced by the deformed and small scales, meanwhile the large cone-like scales (tubercles: defined in Fig. 3 in this report) were not regenerated on the tail or trunk [3–5]. Another study on the tail regeneration after autotomy reported the incomplete recovery of the *de-novo* pigmentation in the newly regenerated tail [3, 4], but the coloration was somewhat blurred and different from the original patterns [3, 4]. The skin regeneration was also studied by experimental excision [5], it was reported that the skin also underwent the scar-free regeneration and restored rather complex patterns of coloration. And interestingly, the regenerated skin close to the pre-existent pigments was also slightly pigmented (see Fig. 2 in [5]), implying that the pigment cells would migrate to the lesion site from the nearby pigmented spots.

In clinical situations, for a variety of diseases are known for *E. macularius*, and surgerical resection is often an inevitable medical option [6]. In the course of surgery, the spotted portion of the skin is sometimes removed due to severe dermal damage or subcutaneous adhesion as will be shown in the present report. Inspired by a series of the previous reports [3–5], the author proposed to preserve a portion of the pigmented skin edge to regenerate as much the same pigment patterns as possible in a case in which the tumor should be excised together with the ulcerated.

## Case presentation

A four-year-old female leopard gecko *E. macularius* suffered from a subcutaneous tumor　in the occipito-pterional portion behind its right eye. The subject weighed 35 g at the date of surgery and was fed daily with commercial mealworms supplemented with calcium and vitamin D powder and housed in a 60 (width) x 30 (depth) x 35 (height) cm^3^ acryl cage, equipped with a floor panel heater, calcium-containing sand bedding, and a 75 W night-light lamp throughout the observation. Room temperature was maintained at 27–30 °C, with the humidity at 60–80 %.

### Surgical operation

**Fig. 1:**
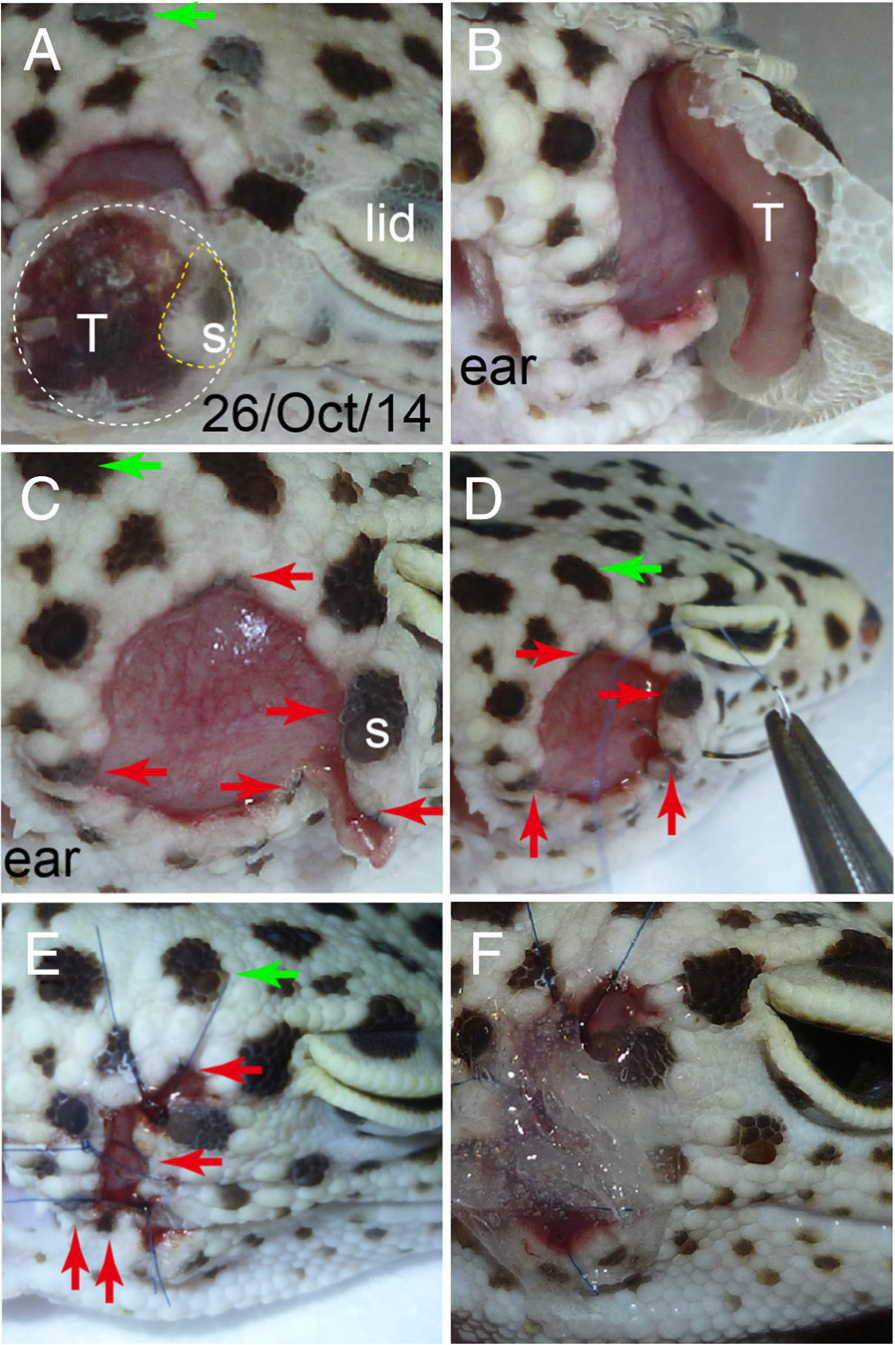
Photographic review of the pigment regeneration. **a** The cracked and ulcerated skin due to the tumor observed from the occipital direction. The white dotted circle indicates the rim of the tumor (T). The *yellow dotted line* indicates the skin flap. A *green arrow* pointing an ecliptic spot is used as a landmark to identify the orientation of the other spots in all the panels hereafter. **b** Appearance of the tumor attached to the flapped skin together with the necrotic tissue. **c** The subcutaneous appearance after the tumor excision. Red arrows, in all the figures hereafter, indicate the preserved dermal spots in the skin margin. **d**, **e** The remaining skin flap was stretched and sutured to the remaining caudal skin rim with strong tension. **f** The scar was covered with ointment containing gentamicin

Upon first inspection, the tumor mass (T in Fig. 1a and b) was palpable, motile, and smooth but had already developed to an ulcerated lesion. The appetite, activity and consciousness level were normal. The wound was bleeding, and the rear edge of the tumor was cracked and fused to the molting skin (Fig. 1a). Thus, an immediate surgical resection was performed under general anesthesia. A mixture of the following anesthetics was used: 8.0 mL midazolam (Dormicam, Astellas Pharma Inc., Tokyo, Japan), 1.5 mL medetomidine hydrochloride and 0.4 mL butorphanol tartrate (Domitol and Vetorphale, Meiji Seika Pharma Co., Ltd., Japan) was adjusted to 30 mL with normal saline. Prior to the operation, the patient was calmed in a dark box, and 100 μL of the anesthetic mixture was slowly injected subcutaneously twice within ten minutes (200 μL total) into the lateral part of the right femur using a 30-G needle (Terumo, Japan).

The incision was made from the crack at the outer perimeter of the ulcerated skin at the occipito-pterional area (Fig. 1b). The skin flap was partially trimmed with scissors to preserve five pigmented regions in the margin (red arrows; Fig. 1c). The isolated mass was easily enucleated. Note that no communicating fistula was found towards the ocular region (Fig. 1c). The wound was rinsed twice with saline and sutured using a 14 mm curved needle with a 7–0 nylon string (Nazme Seisakusho Co., Ltd., Japan).

The exposed sub-dermal area (E in Fig. 1c) was about 46 mm^2^ large, and the remaining skin flap (s in Fig. 1a) was about 6 mm^2^ large without tension. Consequently, the skin flap was sutured with a sufficient margin from the edge of the incision (Fig. 1d) because the shortened skin needed extensive stretching to cover as much lesion area as possible (Fig. 1e). The uncovered wound (about 2 mm^2^) was plastered with artificial ointment containing Vaseline, saline, and gentamicin (50 μg/mL) to avoid infection and dehydration (Fig. 1f).

**Fig. 2:**
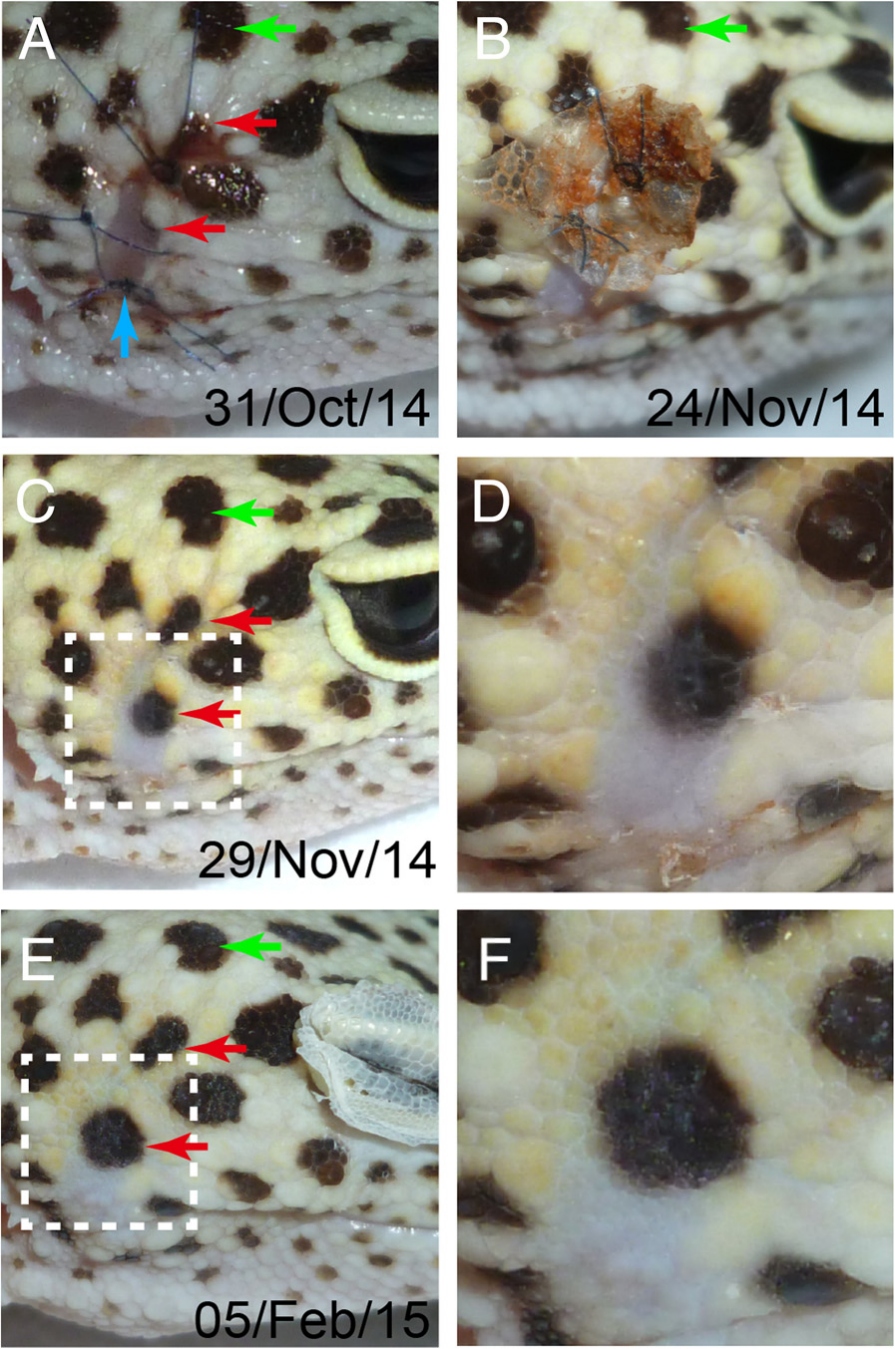
The time-course of the regeneration of the skin. **a** The scar at post-operative day 5. Newly regenerated dermis was observed in the open wound cleft (*blue arrow*). Two out of the four remaining pigmented scales were visible in this panel (*red arrows*). The glistening appearance was due to the remaining ointment. **b** The first molting a month after the surgery. **c** The large and clear spots reappeared in a post-operative month (red arrows). **d** Close-up view of the dotted square area in (**c**). **e** The spots became larger and more vivid at 4 post-operative months. **f** Close-up view of the dotted square area in (**e**). Green arrow is the same landmark scale shown in Fig. 1a

A total of 100 μL gentamicin solution (100 μg/mL) was also orally administered twice a day for one post-operative week. The post-operative course was stable and uneventful. The dermal wound epithelium was rapidly regenerated by post-operative day 5 as previously reported [5], but the pigmented spots had not changed much (Fig. 2a). One month following the surgery, the subject underwent the first post-operative molting (Fig. 2b), and the pigmented spots had grown from the remaining pigments with a little bit blurred perimeter (Fig. 2c and d). In the four months since the surgery, the regenerated spots looked almost undistinguishable from the pre-existent spots with a rather vivid border, and the epidermal scales with yellow coloration were restored (Fig. 2e and f). The delayed yellowish coloration should be a general recovery course of the epidermis in *E. macularius* [4].

**Fig. 3:**
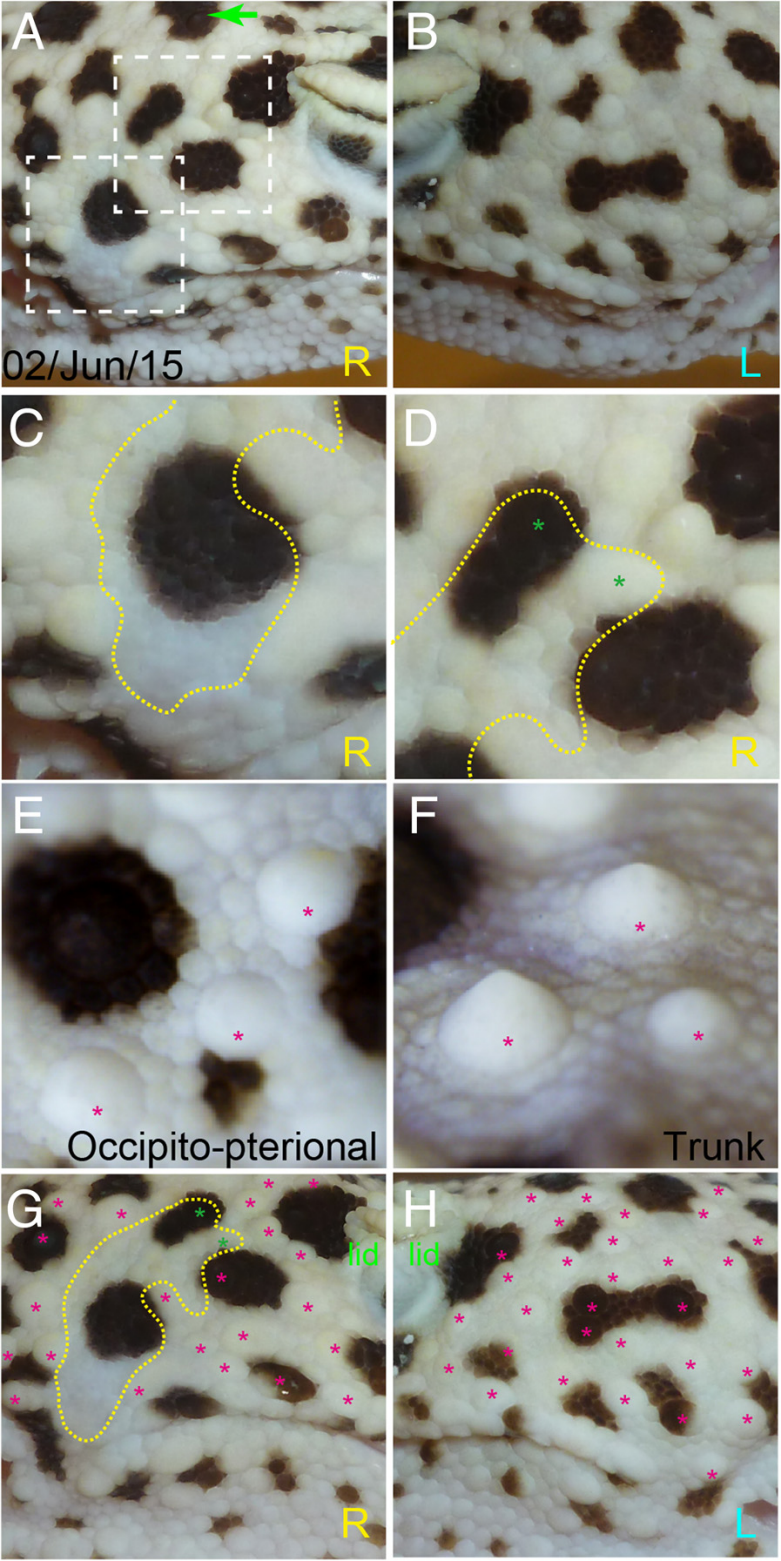
The close observation of the regenerated skin. **a** The regenerated skin at post-operative month 7. **b** The un-operated side on the same date. **c** and **d** Close-up views of the regenerated scales in the dotted squares in (**a**). **e** and **f** Magnified photos of representative scales in occipito-pterional (**e**) and trunk (**f**) regions. **g** and **h** The distribution patterns of the tubercles in the operated side (**g**) and the intact side (**h**). The pigmented spots became larger and more vivid in four post-operative months. *Green asterisks* in (**d**) and (**g**) indicate the regenerated tubercles. *Pink asterisks* in (**e**)-(**h**) are the intact tubercles. The colored letters indicates as follows: R, right; L, left. The green arrow is the same landmark scale shown in Fig. 1a

Followed up for three more months, there were no more changes in the wound healing process in the operated area (Fig. 3a). The newly pigmented spots were distributed evenly and the appearance looked rather natural compared to the un-operated side (Fig. 3b). The regenerated area surrounded by the imaginary borders of the surgical margin (yellow dotted line: Fig. 3c, d and g) was about 20 mm^2^. On higher magnification, the skin in *E. macularius* is tessellated with two different types of scales (Fig. 3e and f): small polygonal scales and cone-like large interspersing scales with round perimeters (defined as tubercles). The tubercles are rather flattened at the occipito-pterional region (pink asterisks in Fig.ure 3e) compared to those on the trunk (pink asterisks in Fig. 3f). The regenerated skin comprised of the small mosaic scales with no obvious scar as reported previously [3–5]. However, much of the large cone-like tubercles were not regenerated but only sparsely located in the operated side (pink asterisks in Fig. 3g), whereas such tubercles were evenly distributed in the intact side (pink asterisks in Fig. 3h). But, it is noteworthy that two tubercles were newly formed in the dorsal area of the operated region (green asterisks in Fig. 3d and g: compare with Fig. 1c).

## Discussion

Although a large portion of the dermal tissues was completely lost and disconnected because of the surgical operation, the regenerated spots grew rapidly and re-localized again evenly and clearly (Fig. 3a). These new spots were also equally away from the pre-existing adjacent spots, so the potential of the color restoration might be influenced by the remaining chromatophores in the vicinity. In addition, a few tubercles were restored in the dorsal part of the occipito-pterional portion, but not near the corner of the mouth (Fig. 3d and g). Considering that the tubercles on the tail or trunk of *E. macularius* do not regenerate [3–5]**,** the skin regeneration at the occipito-pterional portion behind the eye is rather unique. The mechanism how the tubercles could regenerate at the occipito-pterional portion is unclear, but the regeneration potential might differ between the rostral and caudal parts of the body of *E. macularius.* Importantly, not all the reptilian species have the remarkable potentials of scar-free regeneration like *E. macularius* [7]. So, the pattern restoration after the skin damage needs further investigation across the species.

## Conclusion

The surgical suture of the skin flap after removal of the ulcerated margins resulted in the scar-free regeneration of the scales and the pigmented spots. And the pigmented spots of the remaining skin close to the lesion site might be a source of the regenerated spots.

## Abbreviation

*E. macularius*, *Eublepharis macularius*

## References

[1] De Vosjoli P., Ron Tremper R., Klingenberg R. The Herpetoculture of Leopard Geckos. California: Advanced Visions Inc.; 2005.

[2] Carlson BM. Principles of regenerative biology. Massachusetts: Academic Press-Elsevier Ltd; 2007.

[3] McLean KE, Vickaryous MK (2011). A novel amniote model of epimorphic regeneration: the leopard gecko, Eublepharis macularius. BMC Dev Biol.

[4] Delorme SL, Lungu IM, Vickaryous MK (2012). Scar-free wound healing and regeneration following tail loss in the leopard gecko, Eublepharis macularius. Anat Rec (Hoboken).

[5] Peacock HM, Gilbert EA, Vickaryous MK (2015). Scar-free cutaneous wound healing in the leopard gecko, Eublepharis macularius. J Anat.

[6] Alworth LC, Hernandez SM, Divers SJ (2011). Laboratory reptile surgery: principles and techniques. J Am Assoc Lab Anim Sci.

[7] Wu P, Alibardi L, Chuong CM (2014). Regeneration of reptilian scales after wounding: neogenesis, regional difference, and molecular modules. Regeneration (Oxf).

